# Non-Assembled ORF2 Capsid Protein of Porcine Circovirus 2b Does Not Confer Protective Immunity

**DOI:** 10.3390/pathogens10091161

**Published:** 2021-09-09

**Authors:** Flavia Guarneri, Matteo Tonni, Giuseppe Sarli, Maria Beatrice Boniotti, Davide Lelli, Ilaria Barbieri, Giulia D’Annunzio, Giovanni Loris Alborali, Barbara Bacci, Massimo Amadori

**Affiliations:** 1 Laboratory of Animal Welfare, Clinical Chemistry and Veterinary Immunology, Istituto Zooprofilattico Sperimentale Della Lombardia e Dell’emilia Romagna, via A. Bianchi 9, 25124 Brescia, Italy; flavia.guarneri@gmail.com; 2 Diagnostic Laboratory, Istituto Zooprofilattico Sperimentale Della Lombardia e Dell’emilia Romagna, via A. Bianchi 9, 25124 Brescia, Italy; matteotonni89@gmail.com (M.T.); giovanni.alborali@izsler.it (G.L.A.); 3 Department of Veterinary Medical Sciences, University of Bologna, 40126 Bologna, Italy; giuseppe.sarli@unibo.it (G.S.); giulia.dannunzio2@unibo.it (G.D.); barbara.bacci@unibo.it (B.B.); 4 Genomics Department, Istituto Zooprofilattico Sperimentale Della Lombardia e Dell’emilia Romagna, via Antonio Bianchi 7/9, 25124 Brescia, Italy; mariabeatrice.boniotti@izsler.it (M.B.B.); ilaria.barbieri@izsler.it (I.B.); 5 Virology Department, Istituto Zooprofilattico Sperimentale Della Lombardia e Dell’emilia Romagna, via Antonio Bianchi 7/9, 25124 Brescia, Italy; davide.lelli@izsler.it

**Keywords:** pig, Porcine Circovirus 2, ORF2 capsid protein, vaccine, protection

## Abstract

Porcine Circovirus 2 (PCV2) vaccines are based on either inactivated whole virion, or recombinant ORF2 capsid protein assembled into Virus-like Particles (VLPs). No data are available about the immunizing properties of free, non-assembled capsid protein. To investigate this issue, ORF2 of a reference PCV2b strain was expressed in a Baculovirus-based expression system without assembly into VLPs. The free purified protein was formulated into an oil vaccine at three distinct Ag payloads: 10.8/3.6/1.2 micrograms/dose. Each dose was injected intramuscularly into five, 37-day old piglets, carefully matched for maternally-derived antibody. Five control piglets were injected with sterile PBS in oil adjuvant. Twenty-eight days later, all the pigs were challenged intranasally with 10^5.3^ TCID_50_ of PCV2b strain DV6503. After challenge infection, all the pigs remained in good clinical conditions. The recombinant vaccine did not induce significant antibody and PCV2-specific IFN-γ responses. ELISPOT and lymphocyte proliferation data confirmed poor induction of cell-mediated immunity. In terms of PCV2 viremia, there was no significant difference between vaccinated and control animals. The histological data indicated the absence of a detectable viral load and of PCVAD lesions in both vaccinated and control animals, as well as of histiocytes and multi-nucleated giant cells. We conclude that free, non-assembled ORF2 capsid protein does not induce protective immunity.

## 1. Introduction

The Porcine Circovirus Associated Disease (PCVAD) complex includes a series of devasting syndromes [1]. PCVAD is sustained by Porcine Circovirus 2 (PCV2), a small, non-enveloped virus of the family Circoviridae [2]. PCVAD is triggered by PCV2 in association with other pathogens, as well as environmental, non-infectious stressors [3].

Plenty of nucleotide substitutions underlay the rapid emergence of new PCV2 strains worldwide. This way, in addition to the two original main groups designed as genotype “a” (PCV2a) and “b” (PCV2b), further genotypes later emerged in the field (PCV2c, PCV2d, PCV2e) [4,5].

Several vaccines were developed to reduce PCV2 infection and PCVAD occurrence. Importantly, almost all commercial vaccines are based on PCV2a, which shows cross-protection against the other PCV2 genotypes [6]. In practice, multiple PCV2 genotypes are included in one single serotype [7]. In this respect, our previous study [8] showed high immunizing properties of an inactivated, whole virion PCV2a vaccine, whereas a PCV2b vaccine with the same formulation was less immunogenic, on the basis of the much greater antigen mass needed to prevent post-challenge viremia [9].

Interestingly, all the current PCV2 vaccines are based on either inactivated whole viral particles, or ORF2 capsid (Cap) protein, assembled into Virus-like Particles (VLPs) [4]. Since no data is available about the immunizing properties of non-assembled Cap protein, the latter was tested in our study as a possible candidate antigen for PCV2 vaccines. Our working hypothesis was that the native configuration of the PCV2 capsid protein could affect its immunogenicity.

## 2. Results

### 2.1. No PCVAD Was Observed after Challenge and No Virological Protection Was Induced by the Experimental PCV2 Vaccine

Piglets enrolled were in good health conditions. On the day after vaccination, piglets inoculated with the highest Ag dose (10.8 micrograms) and the control ones seemed somewhat depressed and reduced feed intake. Since the two rooms had an unfavorable northbound exposure, the floors were extensively supplemented with straw. The symptoms were over after three and four days in the control and 10.8 microgram groups, respectively. On day 1 after vaccination, a FANS drug (Findol, Ceva Vetem SpA, Italy) was injected into piglet 060 (controls) showing slight lameness, which fully subsided after 3 days. On day 4 after vaccination, Cobactan (Msd Animal Health Srl) was injected into piglet 057 (controls) because of respiratory symptoms; this piglet was found dead on the following day. Post mortem examination revealed embolic pneumonia, from which *Pasteurella multocida* was isolated. No other pigs presented clinical symptoms over the whole study. No signs of PCVAD were observed after challenge infection, in either control or vaccine groups. Also, the weight gains observed after challenge were not significantly different between groups and showed no correlation with the Cap vaccine doses (Supplementary Figure S1). Viremia after challenge was observed in all the control and vaccinated pigs, starting at DPI 14 (Table 1). Viremia was highest at DPI 14 in Group C (1.2 micrograms/dose), in which it significantly declined until DPI 28 (*p* < 0.001). On the contrary, no significant decay of viremia after DPI 14 was observed in the other groups under study. Also, no significant difference was observed between the viremia levels of the four experimental groups at any time point after infection.

### 2.2. The Recombinant Cap Vaccine Did Not Induce a Significant Antibody Response to PCV2

**Figure 1:**
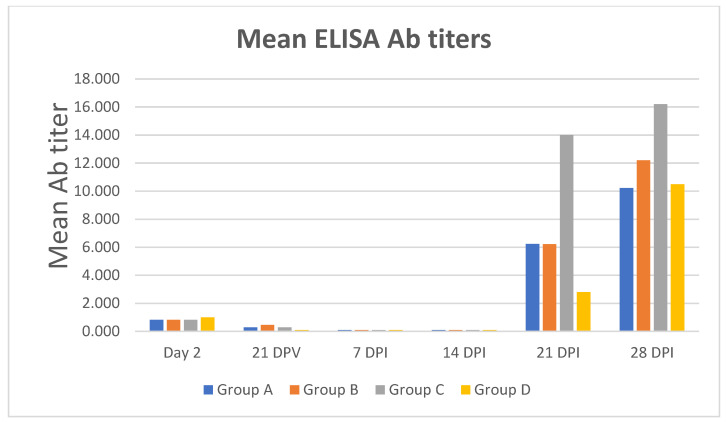
Time-course of the mean ELISA Ab titers of ORF2 antigen-vaccinated (Groups A–C) and control pigs (Group D). No significant Ab response was evidenced at 21 DPV. After challenge infection, there was a late seroconversion in all the groups at 21 DPI. Day 2: day 2 after the arrival at the isolation facilities. Group A: 10.8 μg ORF2 antigen/dose. Group B: 3.6 μg ORF2 antigen/dose. Group C: 1.2 μg ORF2 antigen/dose. Group D: placebo.

The results obtained by ELISA are shown in Figure 1. There was a regular, homogeneous drop of maternally-derived antibody (MDA) titers in all the control animals, whereas some vaccinated animals maintained their MDA titers until 21 DPV, mainly in the 3.6-microgram group (Table 2). Seroconversion after PCV2 infection appeared at 21 DPI in all the groups under study (Figure 1), after the onset of PCV2 viremia at 14 DPI (Table 1).

As for neutralizing antibody, we carried out first a screening of sera at 21 DPV. Unexpectedly, all the sera tested positive at the same titer (≥1:16). Later on, SN titers varied between 1:20 and ≥1:40, with no significant difference between groups. We also checked the number of FFUs (non-neutralized fraction) at the end-point of each titration. Once again, no significant difference between groups was revealed from DPV 21 to DPI 21. On the contrary, the non-neutralized fraction was significantly greater in the control group at DPI 28 (*p* = 0.029); such a difference was more pronounced between control and 1.2-μg group (Šidák post test, *p* = 0.09, tendency).

### 2.3. Poor Cell-Mediated Immune Responses to PCV2 Were Induced by the Cap Vaccine

The PCV2-specific IFN-γ response in whole blood samples to both inactivated PCV2b and recombinant Cap is shown in Table 3. The PCV2 virion-specific IFN-γ response at 21 DPV was not different between control (maternal immunity) and vaccinated animals in terms of prevalence and height. At the same time, the highest Ag payload (Group A, 10.8 μg) induced 3 positive reactions out of five to recombinant ORF2 antigen, as opposed to the other groups under study (P 0.157, NS). Most important, no PCV2 virion-specific IFN-γ response was observed in vaccinated animals after DPV 21.

The IFN-γ response to PCV2 was further dissected in our flow cytometry assay for IFN-γ positive T lymphocytes in PBMC of three pigs per group ( Supplementary Figure S2). The number of positive responses was reckoned at each time point for CD4, CD8α, CD8β, and γ/δ T cells, respectively. There was a significantly higher number of responses in Group D (controls) at 21 DPV (*p* = 0.020), as well as in Groups B and D at 21 DPI (*p* = 0.023). A complete suppression of the IFN-γ responses in all the groups was observed at 28 DPI.

**Figure 2:**
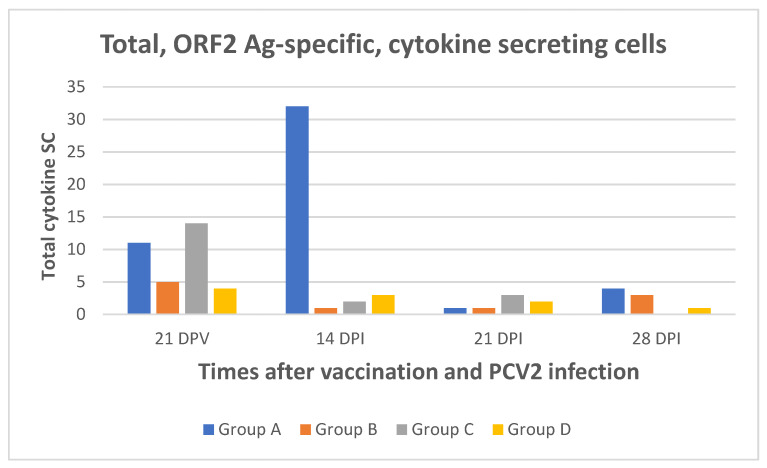
ELISPOT and FLUOROSPOT assays. Total cumulative number of ORF2 antigen-specific, IFN-γ, IL-2, and TNF-α SC/2 × 10^5^ PBMC in ELISPOT assays (21 DPV, 14 and 21 DPI), and of IFN-γ SC only in Fluorospot assays (28 DPI). ELISPOT and FLUOROSPOT assays were carried out on the same 3 pigs/group at the indicated times. The observed differences at 14 DPI were significant (p = 0.042). Group A: 10.8 μg ORF2 antigen/dose. Group B: 3.6 μg ORF2 antigen/dose. Group C: 1.2 μg ORF2 antigen/dose. Group D: placebo.

The same samples were also submitted to ORF2 antigen-specific ELISPOT and FLUOROSPOT assays (Figure 2). In agreement with the aforementioned results, very few ORF2 antigen-specific SC were revealed for the 3 cytokines under study at 21 DPV, with no significant difference between vaccinated and control animals. A significantly higher prevalence was shown instead in Group A at 14 DPI (*p* = 0.042), as opposed to 21 and 28 DPI (Figure 2). Within the four experimental groups, no significant difference was shown between the different time points (ANOVA for repeated measures, *p* > 0.1). Also, no significant Cap-specific response was observed in terms of IL-2/IFN-γ double-positive SC at any time point.

The results of the BrDU assay were in agreement with the above findings (Table 4). Only three out of fifteen vaccinated pigs (Groups A, B, C) were weakly test-positive at 21 DPV. Interestingly, a response was evident at 7 DPI in control and 1.2-μg groups, as opposed to pigs given a higher Ag payload. The response was completely suppressed at 21 DPI and partly resumed at 28 DPI only in the 10.8 and 3.6 μg groups.

### 2.4. NK Assays

**Figure 3:**
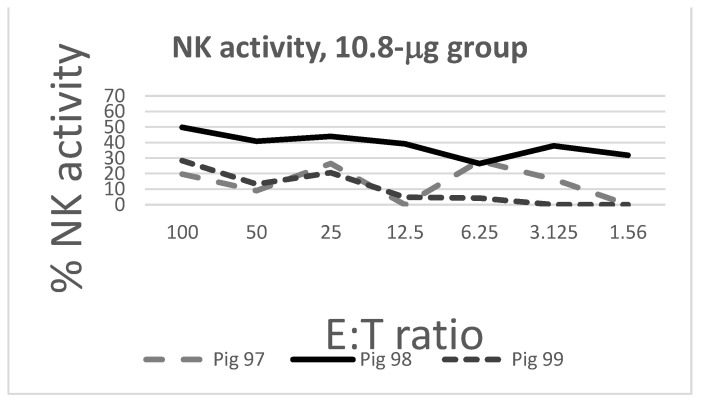

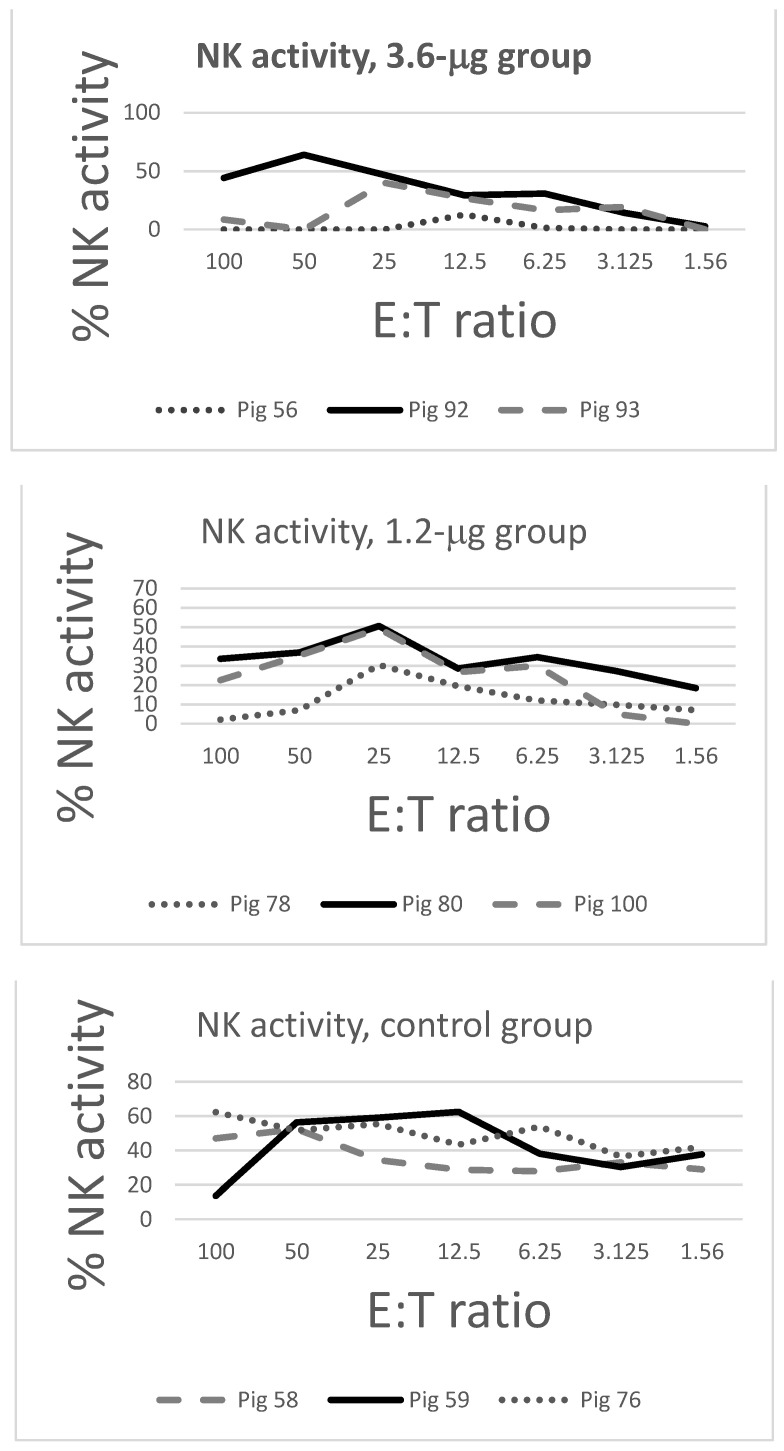
NK activity of tracheobronchial lymph node cells against K-562 target cells. Three pigs per group were included in this assay. The NK activity of control pigs (group D) was higher and more homogeneous (lesser SD) at E:T ratios 50, 3.125, and 1.56, with a significant difference at E:T ratio 1.56 (*p* = 0.019).

The NK activity of tracheobronchial lymph node cells of pigs is shown in Figure 3. The NK activity of control pigs (Group D) was higher and more homogeneous (lesser SD) at E:T ratios 50, 3.125, and 1.56, with a significant difference between vaccinated and control pigs at E:T ratio 1.56 (*p* = 0.019).

### 2.5. Histology

No change indicative of PCVAD was apparent by histology. Lymphoid tissues exhibited various degrees of hyperplasia. The mean and median values of hyperplasia in the lymphoid tissues of the four groups are reported in Supplementary Figure S3, with no significant variation among groups.

Immunohistochemistry did not reveal any detectable PCV2 antigen in all the lymphoid tissues under study (data not shown).

## 3. Discussion

Ever since 2006, PCV2 vaccines have been developed and successfully tested on the basis of the PCV2a genotype. Such vaccines are based on either inactivated, complete viral particles, or capsid ORF2 protein assembled into VLPs [4]. On the basis of these results, we set out to investigate the possible immunizing properties of non-assembled ORF2 protein to fully grasp the importance of the PCV2 capsid structure.

Our results unambiguously show that non-assembled ORF2 protein does not induce protective immunity in pigs even at much higher doses, compared with conventional, whole virus, inactivated vaccines (quite effective at doses way below 1 μg virion antigen) [8,9].

Retrospectively, although the response conferred by commercial vaccines based on ORF2 as VLPs has been widely described, the inclusion of a VLPs-vaccinated group as positive control would have been desirable on the basis of our findings. Please notice however that our working hypothesis did not imply a total failure of our experimental vaccines. Therefore, it was our understanding that possible differences in immunizing properties should be best revealed by quantal potency assays on pigs of the same age and genetic background, vaccinated in the same thermoneutral season (late summer–autumn). These assays imply the vaccination of test groups with different antigen payloads on a regular log scale, in order to estimate the 50% Protective Dose (PD50) by Probit analysis (see e.g., http://userwww.sfsu.edu/efc/classes/biol710/probit/ProbitAnalysis.pdf, accessed on 28 July 2021).

This seasonal condition could not be implemented because of technical reasons; the authors are aware that the setting of this vaccine trial in winter could represent a possible bias. Please notice, however, that the four rooms were heated by infrared lamps, submitted to stepwise height regulation to prevent excessive exposure to heat in the standing position; straw bedding was employed as isolating material in each room. On the whole, the housing conditions of the pigs under study did not substantially differ from those of most pig herds in the same season. Also, pigs were vaccinated at 37 days of age, compared with 45 in our previous study [9]. As opposed to this latter study, pigs showed moderate levels of PCV2-specific neutralizing antibodies at the time of vaccination, whereas the ELISA titers were in the expected range. Therefore, the ratio between neutralizing and ELISA antibodies was higher than previously observed by us in pigs of the same age and genetic background. Accordingly, pigs also showed moderate levels of maternally-derived, cell-mediated immunity, as described in previous studies [10,11]. Please notice that a partial breakdown of immunity to PCV2 had taken place in the herd four months before the birth of the piglets under study, which may have implied a more pronounced transfer of passive immunity in the following months. This aspect is probably correlated with the delayed onset of seroconversion in both vaccinated and control piglets at 21 DPI. Nevertheless, on the basis of published evidence [12], PCV2 vaccines are expected to induce protective immunity despite moderate MDA titers at the time of vaccination. This was not the case. Whereas with 200 to 800 ng of whole virion, inactivated antigens were shown to confer full protection to challenge infection in pigs of the same age and genetic background [8,9], much higher doses of free, recombinant ORF2 product outside the virus capsid structure did not confer any significant protection to experimental challenge. On the basis of the ORF2 sequences of both vaccine and challenge virus, no amino acid differences can account for the lack of protective immunity. Also, the recombinant Cap vaccine was seemingly associated to a worse profile of innate immunity, according to our NK assays (Figure 3).

These findings are definitely in agreement with the poor results of vaccines based on isolated VP1 protein of Foot-and-Mouth Disease virus (FMDV), which highlights the crucial importance of the hycosahedral structure of non-enveloped viruses and native three-dimensional structure of capsid proteins for an effective induction of protective immunity. In practice, the immunogenicity of FMDV VP1 was shown to be several orders of magnitude lower than that of the same protein incorporated in viral particles [13]. Therefore, as in the case of FMDV VP1, the very low immunogenicity of PCV2 ORF2 product might be related to inadequate folding in solution and limited exposure of immunogenic sites to the host’s immune system. This tenet is in agreement with our results with a panel of PCV2-specific, monoclonal antibodies: some of them were only reactive with whole virus in sandwich ELISA, and not with isolated Cap (data not shown). In practice, it is conceivable that free, non-assembled Cap does not adequately present critical conformational epitopes to the immune system. In addition to that, the protein would probably fail to induce cross-presentation of T cell epitopes through MHC I for a CTL response [14], which might be afforded instead by coupling viral proteins to suitable vectors like Heat Shock Proteins [15]. Instead, the virion capsid and VLP structures might be directly suited to cross-presentation through MHC I, in agreement with accumulated data [16,17]. In this respect, cross-presentation of viral peptides may be pivotal to the protective immune response induced by whole virion and VLP PCV2 vaccines. This tenet is supported by the observed protection of VLP-vaccinated pigs even without a detectable Ab response [18]. Also, a clear discrepancy was observed in our study (Table 3) between IFN-γ responses to whole PCV2 virion and to the ORF2 product, respectively, which may imply the lack of cross-presentation of important epitopes to CD8β cytotoxic T cells after vaccination with recombinant, non-assembled ORF2 antigen.

In this conceptual framework, failure of the non-assembled ORF2 product to induce an IFN–γ response in vitro to whole inactivated PCV2 virion was the main immunological marker in this study, since this kind of response had been identified by our group as a main correlate of protection in the PCV2 vaccination and challenge model [8,9]. Interestingly, all the pigs with the lowest levels of viremia (numbers 059/076/056/096/095) also showed IFN-γ responses to the PCV2 ORF2 product (and not to PCV2 virion) between 21 DPV and 7 DPI, with concomitant responses of both CD8β and γ/δ T cells (flow cytometry assay). On the contrary, the proliferation assay at 21 DPV was a poor correlate of protection (compare BrDU and viremia data, Table 2 and Table 4). All the pigs with the lowest levels of viremia (numbers 059/076/056/096/095) also had either delayed seroconversion (at 28 DPI) established by ELISA, or no seroconversion at all (pig 095, Group A). These findings are in agreement with our previous results showing concomitant peaks of PCV2 viremia and ELISA Ab titers [8,9].

As expected, neither PCAVD-specific gross lesion nor microscopic lesions were detected in tissues, and unexpected was the absence of a detectable viral load at least in control and C groups, the latter receiving vaccine with the lowest antigen payload. The absence in the experimental trial of other necessary co-factors following PCV2 infection is the likely explanation for the absence of PCVAD [19], even without vaccine-associated protective immunity. As for failure to detect a viral load in lymphoid tissues by immunohistochemistry, the results can be interpreted as the consequence of the moderate titers of neutralizing antibodies before the start of the trial. Maybe, they were responsible for a reduced ability of the virus to enter the cells; this would result in undetectable amounts of PCV2 in the target organs by immunohistochemistry, regardless of the high viremia levels, as reported in previous trials [20,21].

## 4. Materials and Methods

### 4.1. Virus and Cells

The PCV2b strain DV6503 (Bio Bank Veterinary Resources, IZSLER, Brescia, code VIR RE RSCIC 151) was propagated in Circovirus-free PK15c28 cells (porcine kidney cells, IZSLER cell bank code BS CL 179) as previously described [8]. A cryolysate of PK15c28 cells (mock virus) was set up as well for the assays of cell-mediated immunity, under the same conditions adopted for PCV2 propagation.

Peripheral Blood Mononuclear Cells (PBMC) of pigs were separated by centrifugation of heparinized blood on Histopaque 1.077 (Merck KGaA, Darnstadt, Germany, code 10771-6X100 mL) at 1100× *g*, 25 min, 20 °C, and immediately used in assays of cell-mediated immunity after checking cell viability with the CTL-LDC™ Live/Dead Cell Counting Kit of Cellular Technology Limited (CTL, Cleveland, OH, USA), using an ImmunoSpot S6 Ultimate reader of the same company.

Tracheobronchial lymph nodes were collected during post-mortem examination of twelve pigs and processed as previously described for pig tonsil cells [22]. Aliquots at 5 × 10^6^ cells/mL were frozen at −80 °C in RPMI 1640 medium (50%), Fetal Calf Serum (FCS, 40%), Dimethyl sulfoxide (DMSO, 10%).

### 4.2. Recombinant ORF2 Antigen and Monoclonal Antibodies

ORF2 antigen was produced by Creative Biogene (45-1 Ramsey Road, Shirley, NY 11967, USA) on the basis of a reference PCV2b ORF2 sequence (mRNA Ref seq: AF055394.1; Protein Ref seq: AAC35331.1), using a Baculovirus-based expression system [23] in insect SF9 cells (a clonal isolate of *Spodoptera frugiperda* Sf21 cells). The work included: (1) Synthesis of the PCV2 ORF2 gene sequence. (2) Construction of pFastBac1-PCV2 vector, followed by Baculovirus production. (3) Baculovirus amplification, followed by protein production and purification. ORF2 was designed for expression as free monomeric protein, with no assembly into Virus-like Particles (VLPs). To this purpose, the SP and the Kozak sequences were added to the N-term; meanwhile, a his-tag was added to the C-term, and the final product still contained the tag for purification purposes, but no other supplementary components. The nucleotide and amino acid sequence of the final product is shown in Supplementary Figure S4. For production purposes, recombinant Baculovirus was added to a 1-L bottle containing 200 mL SF9 cells (cell density: 2.0 × 10^6^/mL). After 4 days at 27 °C, the culture was collected and centrifuged, and the supernatant was transferred to sterile 50 mL tubes to perform purification with a Nickel (Ni) metal affinity chromatography column. Cap production was checked by SDS-PAGE and Western Blot with a histidine (his) tag-specific monoclonal antibody. After dialysis against PBS pH 7.4 and sterile filtration, the protein content was verified by the bicinchoninic acid assay (BCA assay) and stored in aliquots at −80 °C. Electron microscopy analyses confirmed the complete absence of VLPs in our recombinant product (see Supplementary Figure S5).

mAb 86D to T cell receptor (TcR) γδ (delta chain-specific), mAb 74-12-4 to porcine CD4, mAb 295-33 to porcine CD8α, and mAb PPT23 to porcine CD8β were kindly provided by A. Saalmüller and W. Gerner (Clinical Immunology, University of Veterinary Medicine, Vienna, Austria).

### 4.3. Vaccine Formulation

The recombinant Cap protein was mixed with the adjuvant of Circovac PCV2 vaccine (Ceva Santé Animale, Libourne, France) at an antigen/adjuvant ratio 1: 2.31. The adjuvant, separately available in the Circovac vaccine package, had proved quite effective in our previous studies on experimental PCV2 vaccines [8,9]. A mock vaccine (placebo) was also prepared by using sterile PBS at the same ratio. Three sets of PCV2 vaccines were prepared, containing 10.8/3.6/1.2 micrograms of Cap protein, respectively, in a 0.5-mL inoculation volume.

### 4.4. Experimental Design

All the animal experiments were conducted at IZSLER, Brescia, Italy, in compliance with the internal Ethical Committee for Animal Experimentation, after receiving a specific Project License (n. 230/2018-PR) issued by the Italian Ministry of Health, in accordance with European Union Guidelines (Directive 2010/63/EU on the protection of animals used for scientific purposes). The animals’ care was in accordance with current institutional guidelines.

**Figure 4:**
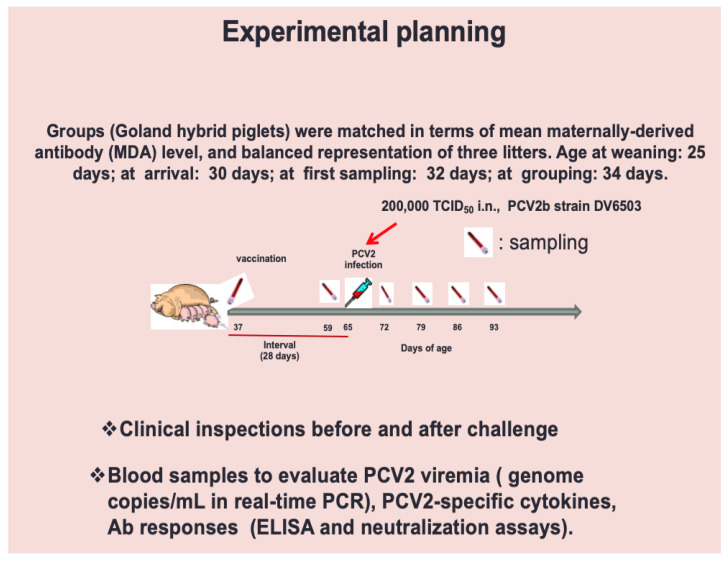
Animal experimental design. The time-course of our study is represented throughout vaccination, samplings and challenge infection. Euthanasia took place later, on two different days, i.e., at 107 (groups C,D) and 108 days of age (groups A,B), to perform necropsy.

The study was carried out on 20 Goland hybrid piglets of 3 litters named White, Green, and Red after their colored ear tag; they were born in December in a farm located in Brescia Province, Italy, with high biosecurity levels. After weaning at 25 days of age, piglets were transferred to IZSLER isolation units five days later and clinically inspected. A first blood sample was collected from each animal two days after the arrival to measure maternally-derived antibody titers to PCV2 by competitive ELISA (see Section 4.5 hereunder). After a further 2 days, pigs were allocated to four groups of 5 subjects each (all of them including at least one pig from each litter) with a balanced distribution of MDA titers (Table 1). After a further three days, three groups of five pigs each (named A, B, and C) were immunized intramuscularly with 0.5 mL of the aforementioned vaccine doses (containing 10.8, 3.6, and 1.2 micrograms of Cap protein, respectively), while group D was treated with 0.5 mL of placebo (see Table 1). Twenty-eight days later all the pigs were challenged intranasally with 2 mL of a suspension containing 10^5.3^ Tissue Culture Infectious Doses 50% (TCID_50_) of the PCV2b strain DV6503. Blood was taken in heparinized vacuum tubes and tubes without anticoagulant at days post vaccination (DPV) 21 and days post infection (DPI) 7, 14, 21, 28. Animals were humanely euthanized on two different days, i.e., 42 (groups C-D) and 43 (groups A-B) DPI, to perform necropsy. The animal experimental design is summarized in Figure 4.

### 4.5. Total and Neutralizing Anti-PCV2 Antibodies

Total PCV2-specific antibodies in serum were measured by competitive ELISA, as previously described [24]. Neutralizing antibodies (NA) in sera and oral fluids were investigated by immunofluorescent staining in PK-15c28 cells as described in our previous study [8]. Titers were expressed as the dilution causing a reduction of the Focus Forming Units (FFUs) ≥ 90%, compared with control wells.

### 4.6. PCV2 DNA in Serum

PCV2 DNA quantification was performed as previously described [25] on serum samples by Real-time quantitative PCR performed after DNA extraction (DNeasy Blood and Tissue Kit, Qiagen, Hilden, Germany). Results were expressed as PCV2 genome copies/mL of serum. 

### 4.7. PCV2-Specific Interferon-γ Release Assay

This assay measures the cell-mediated immune response to PCV2 in heparinized, whole blood samples, and it was carried out as previously described [8]. Purified ORF2 antigen (2 μg/mL final) was also used in separate wells in addition to inactivated PCV2b and mock virus.

### 4.8. Proliferation Assay with Swine PBMC

Immediately after separation, 2 × 10^5^ viable PBMC/well were grown in duplicate in RPMI 1640 medium + 10% heat-inactivated FCS and antibiotics (Penicillin 50 micrograms/mL, Streptomycin 50 micrograms/mL, Amphotericin B 2 micrograms/mL), in 96-well, tissue culture microtiter plates over 7 days in the presence of inactivated PCV2b DV6503, a cryolysate of PK-15c28 cells and growth medium only, respectively. Cell proliferation was measured with the thymidine analog BrdU (5-bromo-2′-deoxyuridine) following its incorporation into newly synthesized DNA and its subsequent detection with an anti-BrdU antibody using a commercial kit (Cell Proliferation ELISA, BrdU, colorimetric, code 11647229001, Roche, Basel, Switzerland), according to the manufacturer’s directions.

### 4.9. PCV2-Specific, IFN γ-Positive T Lymphocytes

PCV2-specific, IFN γ-positive T cells were detected by flow cytometry in a Guava EasyCyte HT flow cytometer using Incyte software (Luminex Corporation, Austin, TX, USA), after in vitro exposure of Peripheral Blood Mononuclear Cells (PBMC) to recombinant PCV2 ORF2 antigen (2 μg/mL), or to medium only (control). Three pigs/group were tested at each sampling for Cap-specific, IFN-γ+ CD4, CD8α, CD8β, and γ/δ T cells, respectively. A time period of 24 h for in vitro re-stimulation was adopted on the basis of previously published data for fresh swine PBMC in assays of cell-mediated immunity to PCV2 [26]. The detailed protocol and the gating strategy are shown in Supplementary Figure S2.

### 4.10. ELISPOT Assays

PBMC samples were also employed in ELISPOT assays for PCV2-specific, IFN-γ, Interleukin (IL)-2, Tumor Necrosis Factor (TNF)-α, as well as IL-2/IFN-γ double-positive secreting cells (SC). The assay was carried out for IFN-γ SC as previously described [27]. For TNF-α and IL-2 SC, we used exactly the same procedure with the mAb couples (capture and detection) of the following kits: Porcine TNF-alpha DuoSet ELISA (cat. DY690B) and Porcine IL-2 DuoSet ELISA (cat. DY652) of R&D Systems (Minneapolis, MN, USA). The dual-color assay was carried out and interpreted as previously described [28]; IFN-γ SC were revealed with the same reagents of the one-color assay; IL-2 SC were revealed using anti-porcine IL-2-Phycoerythrin (PE) Monoclonal Antibody (R&D Systems, cat. IC6521P) and anti-PE, alkaline phosphatase (AP)-conjugated mAb (cod. 600-105-387, TEBU-BIO SrL, Magenta, Italy). Vector AEC (cat. SK-4200) and Vector Blue (Cat. SK-5300) were employed as peroxidase and AP substrates, respectively (Vector Laboratories Inc., Burlingame, CA, USA). Spots were read on an ImmunoSpot S6 Ultimate reader (CTL, Cleveland, OH, USA), using Immunospot software, Double-Color Suite (CTL, Cleveland, OH, USA). PBMC collected at 28 DPI were employed in the one-color FLUOROSPOT assay for IFN-γ SC, using Pig IFN-γ Single-Color FluoroSpot, Capture and Detection kits (cat. PT1000F and PT01, respectively, CTL, Cleveland, OH, USA) according to the manufacturer’s directions. Spots were read on an ImmunoSpot S6 Ultimate reader (CTL, Cleveland, OH, USA), using Immunospot software, FluoroSpot Suite.

### 4.11. Natural Killer (NK) Assay with K-562 Cells

Tracheobronchial lymph node cells were thawed and grown overnight in RPMI 1640 + 10% heat-inactivated FCS + 20 U/mL human recombinant interleukin-2 (HIL-2 RO, Roche, cat. 10799068001, Merck). Next, they were employed in a 4-h NK assay on K-562 cells (human chronic myelogenous leukemia, Biobanking of Veterinary Resources, BVR, Brescia, Italy, code BS TCL 33) [29]. The assay was analyzed using an ImmunoSpot S6 Ultimate reader and the HUMAN NK-TVA™ KIT (Cellular Technology Limited, CTL, Cleveland, OH, USA, code #NK-TVA-5), according to the manufacturer’s directions, as described in our previous paper [27].

### 4.12. Necropsy and Histopathology

Samples of PCV2 target tissues were collected at post-mortem examination: mesenteric, mediastinic and superficial inguinal lymph nodes, spleen, ileum, tonsils, lung, trachea and bronchi, heart, liver, kidney and pancreas. Fixed tissues were processed for histopathologic examination. These were embedded in paraffin wax, sectioned at 4-μm thickness and stained with hematoxylin and eosin. Lymphoid hyperplasia was graded according to a previously published method [27], and tissue sections were selected to perform immunohistochemistry as previously described [9] to assess quantitative PCV2 immunolabeling.

### 4.13. Statistical Analyses

The differences between serum samples in terms of ELISA and neutralizing antibody titers were investigated by one-way ANOVA followed by multiple comparisons between groups using Šidák correction (a further version of the Bonferroni post test). This approach was also applied to the cytokine ELISPOT assays carried out in our study, and to the NK assays.

The prevalence of IFN γ-positive T cells in antigen-stimulated and control cultures (flow cytometry assay) was evaluated on the basis of Fisher’s exact test. To define a positive response, the adopted threshold was a 0.8% difference in prevalence between ORF2 antigen-stimulated and control T cells, which corresponds to *p* < 0.05 for 5000 cells examined on average.

The significance threshold was set at *p* < 0.05. A tendency was declared at *p* < 0.1 (Wizard, version 1.9.48, created by Evan Miller^©^, 2013–2020).

## 5. Conclusions

In conclusion, our study revealed no substantial immunogenicity of PCV2 ORF2 antigen outside the virus capsid structure. In particular, isolated ORF2 product gave rise to very poor Ab and cell-mediated immune responses to PCV2, which could not be discriminated from the passive immunity profiles. Also, the ORF2 antigen-based vaccines failed to induce a PCV2 virion-specific, IFN-γ response in whole blood samples, a marker previously validated as a robust correlate of protection after injection of whole virion, inactivated PCV2 vaccines.

## Figures and Tables

**Table 1:** PCV2 viremia after challenge.

**Group D, Control** **PCV2 Genome Copies/mL**							**Group C, 1.2 μg ORF2/Dose** **PCV2 Genome Copies/mL**						
**Pig**	**DPV0**	**DPV 21**	**DPI 7**	**DPI 14**	**DPI 21**	**DPI 28**	**Pig**	**DPV 0**	**DPV 21**	**DPI 7**	**DPI 14**	**DPI 21**	**DPI 28**
057 *	NEG	-	-	-	-	-	**077**	NEG	NEG	NEG	35.7 × 10^6^	1.4 × 10^6^	3.79 × 10^5^
**058**	NEG	NEG	NEG	4.25 × 10^6^	3.31 × 10^5^	4.48 × 10^4^	**078**	NEG	NEG	NEG	25.3 × 10^6^	1.33 × 10^6^	8.69 × 10^4^
**059**	NEG	NEG	NEG	NEG	17.2 × 10^6^	1.33 × 10^5^	**079**	NEG	NEG	NEG	18.4 × 10^6^	6.13 × 10^5^	3.91 × 10^4^
**060**	NEG	NEG	NEG	2.08 × 10^6^	1.45 × 10^5^	10^4^	**080**	NEG	NEG	NEG	8.86 × 10^3^	3.05 × 10^4^	1.74 × 10^3^
**076**	NEG	NEG	NEG	NEG	4.89 × 10^3^	NEG	**100**	NEG	NEG	NEG	15.6 × 10^6^	2.68 × 10^5^	9.07 × 10^4^
**Group B, 3.6 μg ORF2/Dose** **PCV2 Genome Copies/mL**							**Group A, 10.8 μg ORF2/Dose** **PCV2 Genome Copies/mL**						
**Pig**	**DPV 0**	**DPV 21**	**DPI 7**	**DPI 14**	**DPI 21**	**DPI 28**	**Pig**	**DPV 0**	**DPV 21**	**DPI 7**	**DPI 14**	**DPI 21**	**DPI 28**
**056**	NEG	NEG	NEG	NEG	1.52 × 10^4^	3.74 × 10^4^	**091**	NEG	NEG	NEG	4.89 × 10^5^	8.00 × 10^5^	3.53 × 10^4^
**092**	NEG	NEG	NEG	2.23 × 10^5^	NEG	1.28 × 10^4^	**095**	NEG	NEG	NEG	NEG	5.06 × 10^4^	9.92 × 10^4^
**093**	NEG	NEG	NEG	3.31 × 10^5^	1.34 × 10^4^	2.5 × 10^3^	**097**	NEG	NEG	NEG	2.08 × 10^4^	2.04 × 104	4.29 × 10^3^
**094**	NEG	NEG	NEG	1.51 × 10^5^	8.69 × 10^4^	3.17 × 10^3^	**098**	NEG	NEG	NEG	2.49 × 10^5^	2.76 × 10^5^	2.17 × 10^4^
**096**	NEG	NEG	NEG	NEG	2.02 × 10^5^	8.18 × 10^4^	**099**	NEG	NEG	NEG	3.45 × 10^6^	4.43 × 10^5^	4.69 × 10^4^

Challenge of PCV2-vaccinated and control pigs was carried out at 28 DPV. Sera of PCV2-infected pigs were analyzed by quantitative PCR for ORF2 gene after DNA extraction. Results are expressed as PCV2 genome copies/mL of serum. NEG: undetected PCV2 DNA; * Pig 057 died on DPV 5.

**Table 2:** PCV2 vaccine groups and MDA titers.

**Control, PBS + Adjuvant**				**1.2 μg ORF2/Dose**			
**Group D**				**Group C**			
**Number**	**Litter**	**Starting Titer**	**Titer** **DPV 21**	**Number**	**Litter**	**Starting Titer**	**Titer** **DPV 21**
057 *	White	1/1000	-	**080**	White	1/1000	1/100
**076**	White	1/1000	1/100	**100**	White	1/100	1/100
**059**	Green	1/1000	1/100	**077**	Green	1/1000	1/100
**060**	Green	1/1000	1/100	**078**	Green	1/1000	1/100
**058**	Red	1/1000	1/100	**079**	Red	1/1000	1/1000
**3.6 μg ORF2/Dose**				**10.8 μg ORF2/Dose**			
**Group B**				**Group A**			
**Number**	**Litter**	**Starting Titer**	**Titer** **DPV 21**	**Number**	**Litter**	**Starting Titer**	**Titer** **DPV 21**
**092**	White	1/1000	1/100	**097**	White	1/1000	1/100
**096**	White	1/1000	1/1000	**099**	Green	1/1000	1/100
**093**	Green	1/1000	1/1000	**091**	Red	1/100	1/100
**056**	Red	1/100	1/100	**095**	Red	1/1000	1/1000
**094**	Red	1/1000	1/100	**098**	Red	1/1000	1/100

As a result of the allocation to study groups, at least one pig of the three litters (Red, White, Green, respectively) was represented in each group. Also, there was no significant difference between the mean MDA titers (ELISA Ab assay) of the 4 groups before vaccination. * Pig 057 died from pneumonia on DPV 5.

**Table 3:** In vitro IFN-γ response after PCV2 vaccination and infection.

**Group D, Control** **IFN-γ Response (Ag and DmOD)**						**Group C, 1.2 μg ORF2 Ag/Dose** **IFN-γ Response (Ag and DmOD)**					
**Pig**	**DPV 21**	**DPI 7**	**DPI 14**	**DPI 21**	**DPI 28**	**Pig**	**DPV 21**	**DPI 7**	**DPI 14**	**DPI 21**	**DPI 28**
057 *	-	-	-	-	-	**077**	ORF2:NEG PCV2:NEG	**ORF2: 63** PCV2:NEG	**ORF2: 13** PCV2:NEG	ORF2:NEG PCV2:NEG	ORF2:NEG PCV2:NEG
**058**	**ORF2: 58 PCV2: 25**	ORF2:NEG PCV2:NEG	ORF2:NEG PCV2:NEG	ORF2: NEG PCV2:NEG	ORF2:NEG PCV2:NEG	**078**	ORF2:NEG PCV2:NEG	ORF2:NEG PCV2:NEG	**ORF2: 16** PCV2:NEG	ORF2:NEG PCV2:NEG	**ORF2: 46** PCV2:NEG
**059**	ORF2:NEG **PCV2: 23**	ORF2:NEG **PCV2: 17**	ORF2:NEG PCV2:NEG	ORF2:NEG PCV2:NEG	ORF2:NEG PCV2:NEG	**079**	ORF2:NEG PCV2:NEG	ORF2:NEG PCV2:NEG	ORF2:NEG PCV2:NEG	ORF2:NEG PCV2:NEG	ORF2:NEG PCV2:NEG
**060**	ORF2:NEG PCV2:NEG	ORF2:NEG PCV2:NEG	ORF2:NEG PCV2:NEG	ORF2:NEG PCV2:NEG	**ORF2: 10** PCV2:NEG	**080**	ORF2:NEG PCV2:NEG	ORF2:NEG PCV2:NEG	ORF2:NEG PCV2:NEG	ORF2:NEG PCV2:NEG	ORF2:NEG PCV2:NEG
**076**	ORF2:NEG PCV2:NEG	**ORF2: 22** PCV2:NEG	ORF2:NEG PCV2:NEG	ORF2:NEG PCV2:NEG	ORF2:NEG PCV2:NEG	**100**	ORF2:NEG **PCV2: 20**	ND	ORF2:NEG PCV2:NEG	**ORF2: 90** PCV2:NEG	ORF2:NEG PCV2:NEG
**Group B, 3.6 μg ORF2 Ag/dose** **IFN-γ response (Ag and DmOD)**						**Group A, 10.8 μg ORF2 Ag/dose** **IFN-γ response (Ag and DmOD)**					
**Pig**	**DPV 21**	**DPI 7**	**DPI 14**	**DPI 21**	**DPI 28**	**Pig**	**DPV 21**	**DPI 7**	**DPI 14**	**DPI 21**	**DPI 28**
**056**	ORF2:NEG PCV2:NEG	ORF2:NEG PCV2:NEG	**ORF2: 34** PCV2:NEG	ORF2:NEG PCV2:NEG	ORF2:NEG PCV2:NEG	**091**	**ORF2: 29** PCV2:NEG	**ORF2: 17** PCV2:NEG	ORF2:NEG PCV2:NEG	ORF2:NEG PCV2:NEG	ORF2:NEG PCV2:NEG
**092**	ORF2:NEG PCV2:NEG	ORF2:NEG PCV2:NEG	**ORF2: 10** PCV2:NEG	ORF2:NEG PCV2:NEG	ORF2:NEG PCV2:NEG	**095**	**ORF2:64** PCV2:NEG	**ORF2: 56** PCV2:NEG	**ORF2: 42** PCV2:NEG	ORF2:NEG PCV2:NEG	**ORF2: 16** PCV2:NEG
**093**	ORF2:NEG PCV2:NEG	ORF2:NEG PCV2:NEG	ORF2:NEG PCV2:NEG	ORF2:NEG PCV2:NEG	ORF2:NEG PCV2:NEG	**097**	ORF2:NEG PCV2:NEG	ORF2: 11 PCV2: 30	ORF2: 24 PCV2:NEG	ORF2:NEG PCV2:NEG	ORF2:NEG PCV2:NEG
**094**	ORF2:NEG PCV2:NEG	**ORF2: 36** PCV2:NEG	ORF2:NEG PCV2:NEG	ORF2:NEG PCV2:NEG	ORF2:NEG PCV2:NEG	**098**	ORF2:NEG PCV2:NEG	ORF2:NEG PCV2:NEG	ORF2:NEG PCV2:NEG	**ORF2: 82** PCV2:NEG	**ORF2: 15** PCV2:NEG
**096**	ORF2:NEG PCV2:NEG	**ORF2: 12** PCV2:NEG	ORF2:NEG PCV2:NEG	ORF2:NEG PCV2:NEG	ORF2:NEG PCV2:NEG	**099**	**ORF2: 32** PCV2:NEG	ORF2:NEG PCV2:NEG	**ORF2: 17** PCV2:NEG	ORF2:NEG PCV2:NEG	ORF2:NEG PCV2:NEG

Heparinized, whole blood samples were employed in a PCV2-specific IFN-gamma release assay for recombinant ORF2 antigen (2 μg/mL) and inactivated PCV2b, respectively, at different times after vaccination and experimental infection. Results are expressed in terms of Δ(delta)mOD, i.e., the mOD difference between Ag-stimulated and control wells. The table shows the numerical ΔmOD values. Test-positive and dubious samples are highlighted using bold characters. NEG: test-negative sample (ΔmOD < 10). Dubious samples: ΔmOD ≥ 10, <20. Positive samples: ΔmOD ≥ 20. * Pig 057 died from pneumonia on DPV 5.

**Table 4:** BrDU proliferation assay for PCV2: Delta mOD values.

**Group A: 10.8** **μg ORF2 Ag**						**Group B: 3.6** **μg ORF2 Ag**					
**Pig number**	**21 DPV**	**7 PI**	**14 Pi**	**21 PI**	**28 PI**	**Pig number**	**21 DPV**	**7 PI**	**14 Pi**	**21 PI**	**28 PI**
**091**	NEG	NEG	NEG	NEG	NEG	**056**	NEG	8	NEG	NEG	NEG
**095**	NEG	NEG	NEG	NEG	**148**	**092**	NEG	NEG	NEG	NEG	NEG
**097**	NEG	NEG	NEG	NEG	NEG	**093**	**38**	NEG	NEG	NEG	NEG
**098**	**68**	NEG	NEG	NEG	NEG	**094**	**128**	NEG	NEG	NEG	**144**
**099**	9	NEG	NEG	NEG	**60**	**096**	NEG	NEG	**43**	NEG	15
											
**Group C: 1.2** **μg ORF2 Ag**						**Group D: control**					
**Pig number**	**21 DPV**	**7 PI**	**14 Pi**	**21 PI**	**28 PI**	**Pig number**	**21 DPV**	**7 PI**	**14 Pi**	**21 PI**	**28 PI**
**077**	NEG	**31**	**45**	NEG	NEG	057 *	ND	ND	ND	ND	ND
**078**	1	**52**	NEG	NEG	NEG	**058**	NEG	72	NEG	NEG	NEG
**079**	NEG	NEG	NEG	NEG	NEG	**059**	NEG	33	NEG	NEG	NEG
**080**	NEG	NEG	NEG	NEG	NEG	**060**	NEG	**54**	NEG	NEG	NEG
**100**	**43**	NEG	NEG	NEG	NEG	**076**	NEG	NEG	**40**	NEG	NEG
						* Deceased					

PBMC of each pig (2 × 10^5^ PBMC/0.1 mL/well) in complete growth medium were distributed in six aliquots in tissue culture, 96-well microtiter plates. Two wells were supplemented with 0.1 mL of inactivated PCV2b, a cryolysate of PK-15c28 cells (both of them at the same, pre-established dilution), and growth medium, respectively. Then, PBMC were grown at 37 °C in 5% CO_2_ over 7 days. Cell proliferation was determined on the basis of BrDU incorporation using kit “Cell Proliferation ELISA, BrdU” (Roche), as specified by the manufacturer. Results are expressed in terms of Delta milliOD (mOD) (mean mOD PCV2 wells–mean mOD growth medium or cell cryolysate wells: the higher value of the two was employed for calculations). Delta mOD ≥ 20 was the adopted threshold of test-positive PBMC samples. Test-positive results are highlighted in bold characters. NEG: Delta mOD equal to or <0. ND: not done.

## Data Availability

Data are contained within the article or supplementary material.

## Supplementary Materials

The following are available online at https://www.mdpi.com/article/10.3390/pathogens10091161/s1; Figure S1: Mean weight gains of PCV2 Cap-vaccinated and control pigs. Figure S2: Detection of IFN γ-positive T cell populations: protocol and gating strategy. Figure S3: Grading of hyperplasia in lymphoid tissues. Figure S4: Nucleotide and amino acid sequences of Baculovirus-expressed PCV2 ORF2. Figure S5: electron microscopy analyses of recombinant, purified PCV2 Cap protein.

## References

[1] Segales J. (2012). Porcine circovirus type 2 (PCV2) infections: Clinical signs, pathology and laboratory diagnosis. Virus Res..

[2] Segales J., Olvera A., Grau-Roma L., Charreyre C., Nauwynck H., Larsen L., Dupont K., McCullough K., Ellis J., Krakowka S. (2008). PCV-2 genotype definition and nomenclature. Vet. Rec..

[3] Alarcon P., Velasova M., Mastin A., Nevel A., Stark K.D., Wieland B. (2011). Farm level risk factors associated with severity of post-weaning multi-systemic wasting syndrome. Prev. Vet. Med..

[4] Segales J. (2015). Best practice and future challenges for vaccination against porcine circovirus type 2. Expert Rev. Vaccines.

[5] Harmon K.M., Gauger P.C., Zhang J., Pineyro P.E., Dunn D.D., Chriswell A.J. (2015). Whole-Genome Sequences of Novel Porcine Circovirus Type 2 Viruses Detected in Swine from Mexico and the United States. Genome Announc..

[6] Park K.H., Oh T., Yang S., Cho H., Kang I., Chae C. (2019). Evaluation of a porcine circovirus type 2a (PCV2a) vaccine efficacy against experimental PCV2a, PCV2b, and PCV2d challenge. Vet. Microbiol..

[7] Franzo G., Segales J. (2020). Porcine Circovirus 2 Genotypes, Immunity and Vaccines: Multiple Genotypes but One Single Serotype. Pathogens.

[8] Zanotti C., Martinelli N., Lelli D., Amadori M. (2015). Correlates of Protection Following Vaccination with Inactivated Porcine Circovirus 2 Vaccines. Viral. Immunol..

[9] Guarneri F., Tresoldi E.T., Sarli G., Boniotti M.B., Lelli D., Barbieri I., Bacci B., D’Annunzio G., Amadori M. (2021). Protective immunity in swine induced by Porcine Circovirus 2b inactivated vaccines with different antigen payload. Vet. Microbiol..

[10] Oh Y., Seo H.W., Han K., Park C., Chae C. (2012). Protective effect of the maternally derived porcine circovirus type 2 (PCV2)-specific cellular immune response in piglets by dam vaccination against PCV2 challenge. J. Gen. Virol..

[11] Salmon H., Berri M., Gerdts V., Meurens F. (2009). Humoral and cellular factors of maternal immunity in swine. Dev. Comp. Immunol..

[12] Opriessnig T., Patterson A.R., Elsener J., Meng X.J., Halbur P.G. (2008). Influence of maternal antibodies on efficacy of porcine circovirus type 2 (PCV2) vaccination to protect pigs from experimental infection with PCV2. Clin. Vaccine Immunol..

[13] Sobrino F., Saiz M., Jimenez-Clavero M.A., Nunez J.I., Rosas M.F., Baranowski E., Ley V. (2001). Foot-and-mouth disease virus: A long known virus, but a current threat. Vet. Res..

[14] Colbert J.D., Cruz F.M., Rock K.L. (2020). Cross-presentation of exogenous antigens on MHC I molecules. Curr. Opin. Immunol..

[15] Tsan M.F., Gao B. (2009). Heat shock proteins and immune system. J. Leukoc. Biol..

[16] Grgacic E.V., Anderson D.A. (2006). Virus-like particles: Passport to immune recognition. Methods.

[17] Ludwig C., Wagner R. (2007). Virus-like particles-universal molecular toolboxes. Curr. Opin. Biotechnol..

[18] Koinig H.C., Talker S.C., Stadler M., Ladinig A., Graage R., Ritzmann M., Hennig-Pauka I., Gerner W., Saalmuller A. (2015). PCV2 vaccination induces IFN-gamma/TNF-alpha co-producing T cells with a potential role in protection. Vet. Res..

[19] Opriessnig T., Halbur P.G. (2012). Concurrent infections are important for expression of porcine circovirus associated disease. Virus Res..

[20] Solis Worsfold C., Dardari R., Law S., Eschbaumer M., Nourozieh N., Marshall F., Czub M. (2015). Assessment of neutralizing and non-neutralizing antibody responses against Porcine circovirus 2 in vaccinated and non-vaccinated farmed pigs. J. Gen. Virol.

[21] Fort M., Olvera A., Sibila M., Segales J., Mateu E. (2007). Detection of neutralizing antibodies in postweaning multisystemic wasting syndrome (PMWS)-affected and non-PMWS-affected pigs. Vet. Microbiol..

[22] Razzuoli E., Faggionato E., Dotti S., Villa R., Lombardo T., Boizza L., Ferrari M., Amadori M. (2012). Isolation and culture of pig tonsil lymphocytes. Vet. Immunol. Immunopathol..

[23] Chambers A.C., Aksular M., Graves L.P., Irons S.L., Possee R.D., King L.A. (2018). Overview of the Baculovirus Expression System. Curr. Protoc. Protein Sci..

[24] Sala G., Rigola S., Alborali G.L., Brocchi E., Cordioli P. Development of monoclonal antibodies-based ELISAs for the detection of antibodies against porcine circovirus type 1 and type 2. Proceedings of the 5th International Congress of Veterinary Virology.

[25] Olvera A., Sibila M., Calsamiglia M., Segales J., Domingo M. (2004). Comparison of porcine circovirus type 2 load in serum quantified by a real time PCR in postweaning multisystemic wasting syndrome and porcine dermatitis and nephropathy syndrome naturally affected pigs. J. Virol. Methods.

[26] Steiner E., Balmelli C., Gerber H., Summerfield A., McCullough K. (2009). Cellular adaptive immune response against porcine circovirus type 2 in subclinically infected pigs. BMC Vet. Res..

[27] Guarneri F., Tresoldi E.T., Sarli G., Boniotti M.B., Lelli D., Barbieri I., Bacci B., D’Annunzio G., Amadori M. (2021). Dataset of immune responses induced in swine by an inactivated Porcine Circovirus 2b vaccine. Data Brief..

[28] Boulet S., Ndongala M.L., Peretz Y., Boisvert M.P., Boulassel M.R., Tremblay C., Routy J.P., Sekaly R.P., Bernard N.F. (2007). A dual color ELISPOT method for the simultaneous detection of IL-2 and IFN-gamma HIV-specific immune responses. J. Immunol. Methods.

[29] Welter A., Sundararaman S., Li R., Zhang T., Karulin A.Y., Lehmann A., Naeem V., Roen D.R., Kuerten S., Lehmann P.V. (2018). High-Throughput GLP-Capable Target Cell Visualization Assay for Measuring Cell-Mediated Cytotoxicity. Cells.

