# Supplementary material for: Non-Assembled ORF2 Capsid Protein of Porcine Circovirus 2b Does Not Confer Protective Immunity
Source: Pathogens. 2021 Sep 9;10(9):1161. doi: 10.3390/pathogens10091161 (PMC8466160; doi:10.3390/pathogens10091161)
Supplement: Supplementary file 1 [file pathogens-10-01161-s001.zip › pathogens-1343968-supplementary.pdf]

pathogens

MDPI

Supplementary Material

Non-Assembled ORF2 Capsid Protein of Porcine Circovirus 2b Does Not Confer Protective Immunity

Flavia Guarneri [1], Matteo Tonni [2], Giuseppe Sarli [3], Maria Beatrice Boniotti [4], Davide Lelli [5], Ilaria Barbieri [4], Giulia D'Annunzio [3], Giovanni Loris Alborali [2], Barbara Bacci [3] and Massimo Amadori [1,*]

[1] Laboratory of Animal Welfare, Clinical Chemistry and Veterinary Immunology, Istituto Zooprofilattico Sperimentale Della Lombardia e Dell'emilia Romagna, via A. Bianchi 9, 25124 Brescia, Italy; flavia.guarneri@gmail.com
[2] Diagnostic Laboratory, Istituto Zooprofilattico Sperimentale Della Lombardia e Dell'emilia Romagna, via A. Bianchi 9, 25124 Brescia, Italy; matteotonni89@gmail.com (M.T.); giovanni.alborali@izsler.it (G.L.A.)
[3] Department of Veterinary Medical Sciences, University of Bologna, 40126 Bologna, Italy; giuseppe.sarli@unibo.it (G.S.); giulia.dannunzio2@unibo.it (G.D.); barbara.bacci@unibo.it (B.B.)
[4] Genomics Department, Istituto Zooprofilattico Sperimentale Della Lombardia e Dell'emilia Romagna, via A. Bianchi 9, 25124 Brescia, Italy; mariabeatrice.boniotti@izsler.it (M.B.B.); ilaria.barbieri@izsler.it (I.B.)
[5] Virology Department, Istituto Zooprofilattico Sperimentale Della Lombardia e Dell'emilia Romagna, via Antonio Bianchi 7/9, 25124 Brescia, Italy; davide.lelli@izsler.it
* Correspondence: m_amadori@fastwebnet.it

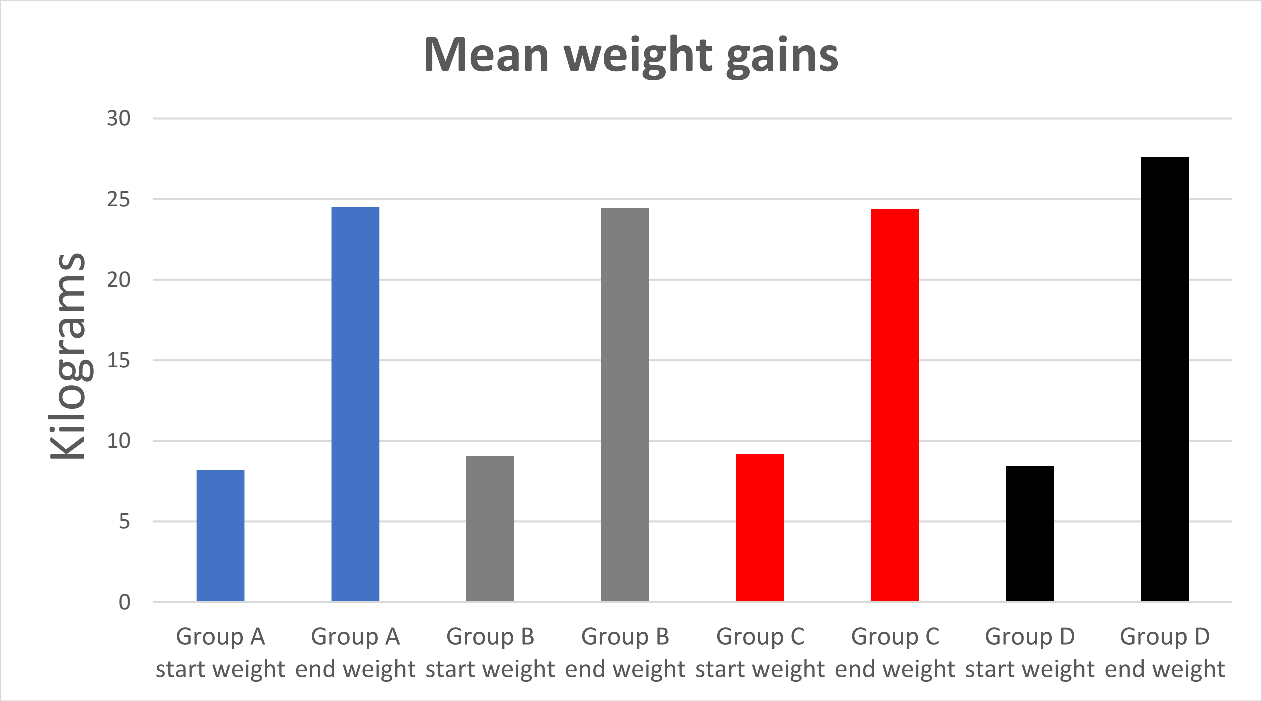

Supplementary Figure S1. Mean weight gains of PCV2 Cap-vaccinated and control pigs. 30-day old Goland piglets of three different litters (White, Green, Red) were transported to our isolation facilities and weighed two days later. After allocation of piglets to the four experimental groups, there was no significant weight difference between groups A, B, C, D. Each pig was weighed once again immediately after slaughter, i.e. after 71 (groups A and B) and 70 days (groups C and D), at DPI 43 and 42, respectively. The daily mean weight gains did not significantly differ between the different groups (P = 0.457). Group A: 10.8 µg ORF2 / dose. Group B: 3.6 µg ORF2 / dose. Group C: 1.2 µg ORF2 / dose. Group D: placebo.

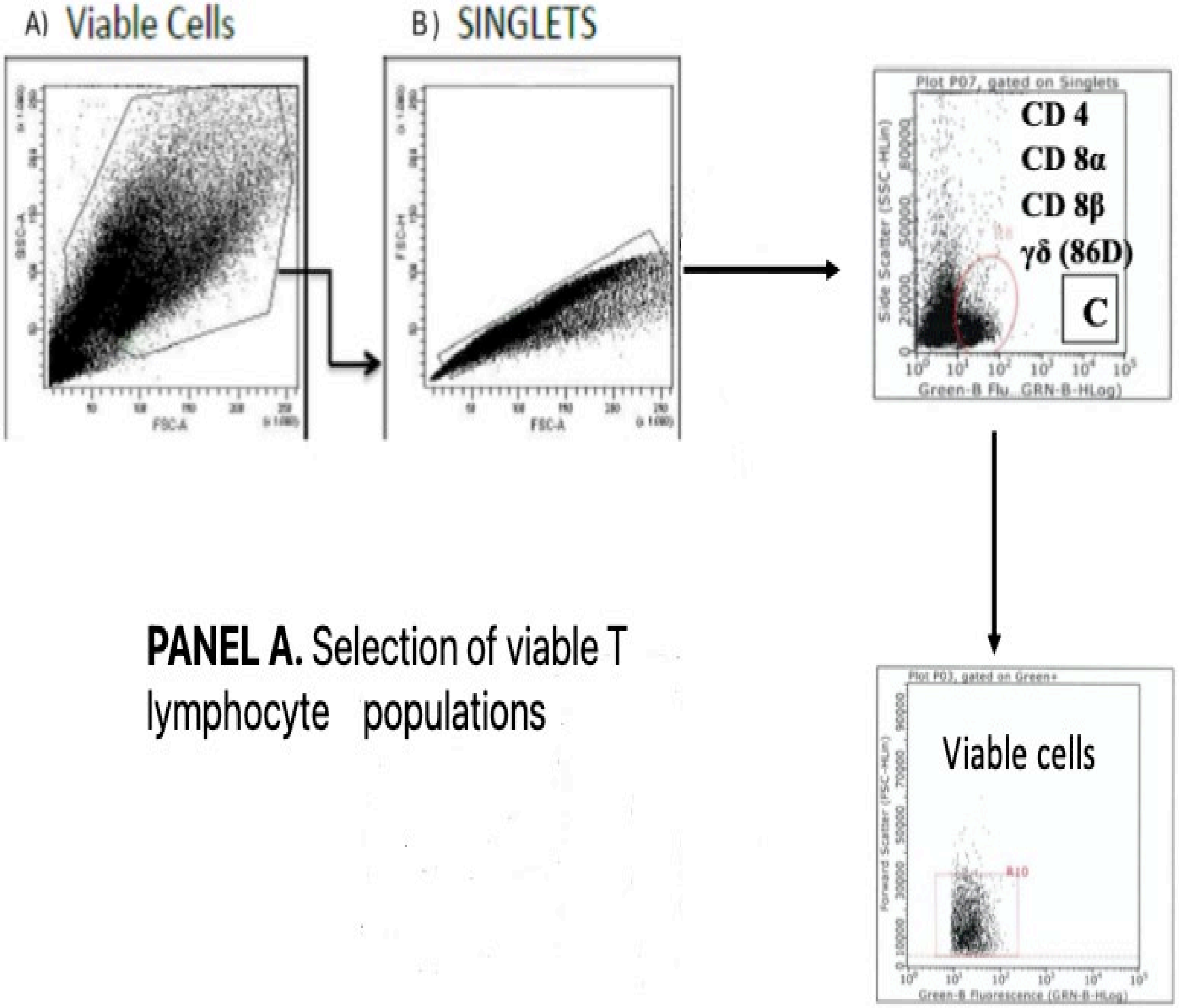

PANEL A. Selection of viable T lymphocyte populations

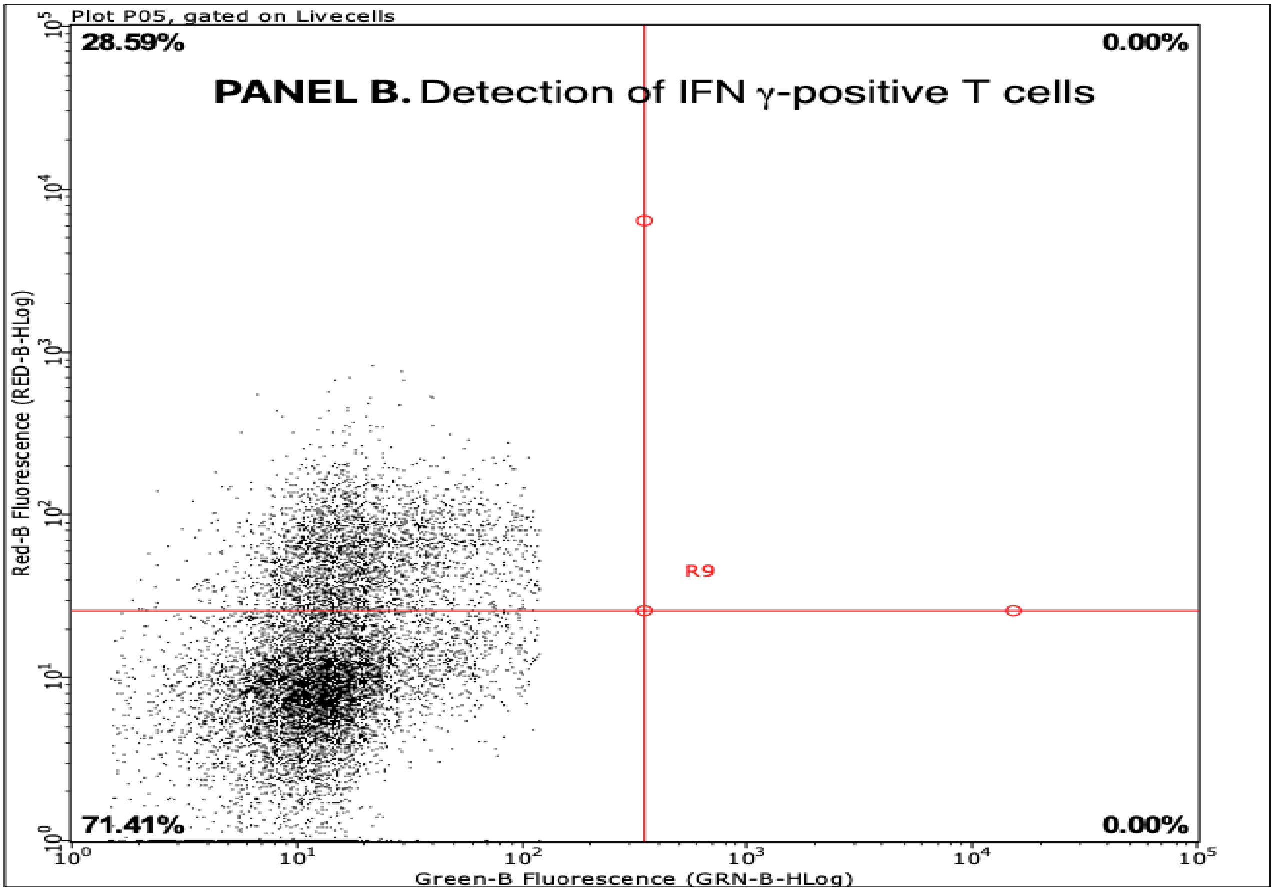

Supplementary Figure S2. Detection of IFN γ-positive T cell populations: protocol and gating strategy. PCV2-specific, IFN-γ-positive T lymphocytes were investigated. PBMC of PCV2-vaccinated and/or infected pigs were grown over 24 hours with and without purified, Baculovirus-expressed PCV2 capsid protein (2 μg/mL). Different aliquots of PCV2 Ag-stimulated and control cells were surface-stained with monoclonal antibodies to porcine CD4, CD8α, CD8β and T cell Receptor (TcR) γ/δ, respectively, followed by Alexa Fluor® 488 F(ab')$_2$ fragment of goat, anti-mouse IgG, IgM (H+L). Next, cells were fixed, permeabilized and stained with mAb CC302 to porcine IFN-γ (BIO-RAD, code MCA 1783), conjugated with PE-Cy5.5 (Lightning-Link PE-Cy5.5 Antibody Labeling Kit, code 761-0010, Novus Biologicals Europe, Abingdon, UK). Cells were gated (Panel A) by a combination of forward and side scatter (A). Next, doublet and multiplet cells were discriminated from single cells in a FSC height / FSC area cytogram (B). After selecting T lymphocyte populations in a SSC x green fluorescence cytogram (C), viable cells were further selected in a FSC x green fluorescence cytogram as previously described [1]. Finally, such viable cells were checked for expression of IFN-γ in a green x red fluorescence cytogram (Panel B). The prevalence of IFN γ-positive in ORF2 antigen/stimulated and control cultures was investigated by Fisher's exact test. To define a positive response, the adopted threshold amounted to a 0.8% difference in prevalence between ORF2 antigen-stimulated and control T cells, which corresponds to P< 0.05 (significance threshold) for 5,000 cells examined on average.

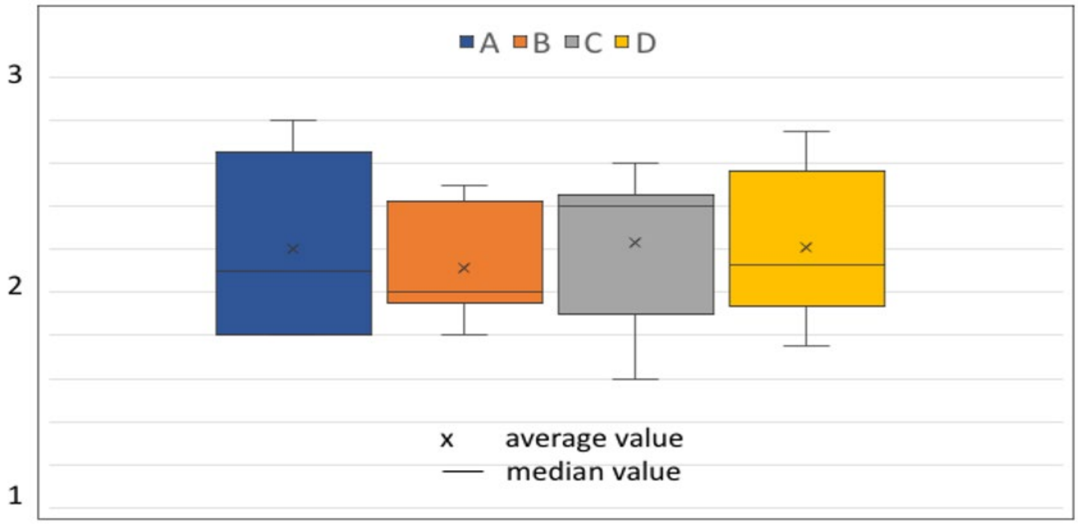

Supplementary Figure S3. Grading of hyperplasia in lymphoid tissues. In each box, the mean (x) and median (line) values of the hyperplasia grade in all the lymphoid sections (tonsil, spleen, ileum, mesenteric, mediastinic and superficial inguinal lymph nodes) of the relevant group are shown. The whiskers indicate SD. Because data are not normally distributed, median is a better indicator of data distribution, and in each group 1 and 3 are the hyperplasia range values (y axis), estimated as previously described [2]. Group A: 10.8 µg ORF2 antigen / dose. Group B: 3.6 µg ORF2 antigen / dose. Group C: 1.2 µg ORF2 antigen / dose. Group D: control.

- BamHI(GGATCC): 1…6 (SP)
- Kozak(GCCACC): 7..12
- Signal Peptide: 13…66
- CDS 67-765 (Sequence optimized with insect systems): 233 amino acids
- His tag: 766-792
- XhoI(CTCGAG): 793…798

Sequence:

GGATCCgccaccATGAAGCTGTGCATCCTGCTGGCTGTGGTGGCCTTCGTGGGTCTGTCCC
TGGGTatgacctacccccgtcgccgctaccgtcgccgtcgtcatcgtcctcgcagccacctgggtcagatcctgcgtcgtcgtccctggctg
gtccaccctcgtcaccgctaccgttggcgccgtaagaacggcatcttcaacacccgcctgagccgcactttcggttacaccgtcaagcgcacta
ctgtgaagacccctagctgggccgtggacatgatgcgtttcaacatcaacgacttcctgcctcctggcggcggctccaaccccagatccgtgcc
tttcgaatactaccgtatccgcaaggtcaaggtggagttctggccctgcagccctatcacccagggtgaccgcggtgtcggtagctccgccgtc
atcctggacgacaacttcgtcactaaggccaccgccctgacttacgacccctacgtcaactacagctcccgtcacactatcacccagcctttctcc
taccactcccgctacttcacccccaagcccgtgctggactccaccatcgactacttccagcccaacaacaagcgcaaccagctgtggctgcgtc
tgcagaccactggcaacgtcgaccacgtgggtctgggcaccgctttcgaaaactccatctacgaccaggagtacaacatccgcgtgaccatgt
acgtgcagttccgcgagttcaacctgaaggaccctcctctgaaccccggcagcCACCACCACCACCATCACTAActcgag

PCV2 ORF2 (amino acid sequence)

MKLCILLAVVAFVGLSLGMTYPRRRYRRRRHRPRSHLGQILRRRPWLVHPRHRYRWRRK
NGIFNTRLSRTFGYTVKRTTVKTPSWAVDMMRFNINDFLPPGGGSNPRSVPFEYYRIRKVK
VEFWPCSPITQGDRGVGSSAVILDDNFVTKATALTYDPYVNYSSRHTITQPFSYHSRYFTPK
PVLDSTIDYFQPNNKRNQLWLRLQTTGNVDHVGLGTAFENSIYDQEYNIRVTMYVQFREF
NLKDPPLNPGSHHHHHH

Supplementary Figure S4. Nucleotide and amino acid sequences of Baculovirus-expressed PCV2 ORF2. The different parts of recombinant ORF2 gene and protein are highlighted using colored text. Also, lower and upper case letters are used to ease the recognition of each component. TAA (yellow): stop codon before the XhoI restriction site.

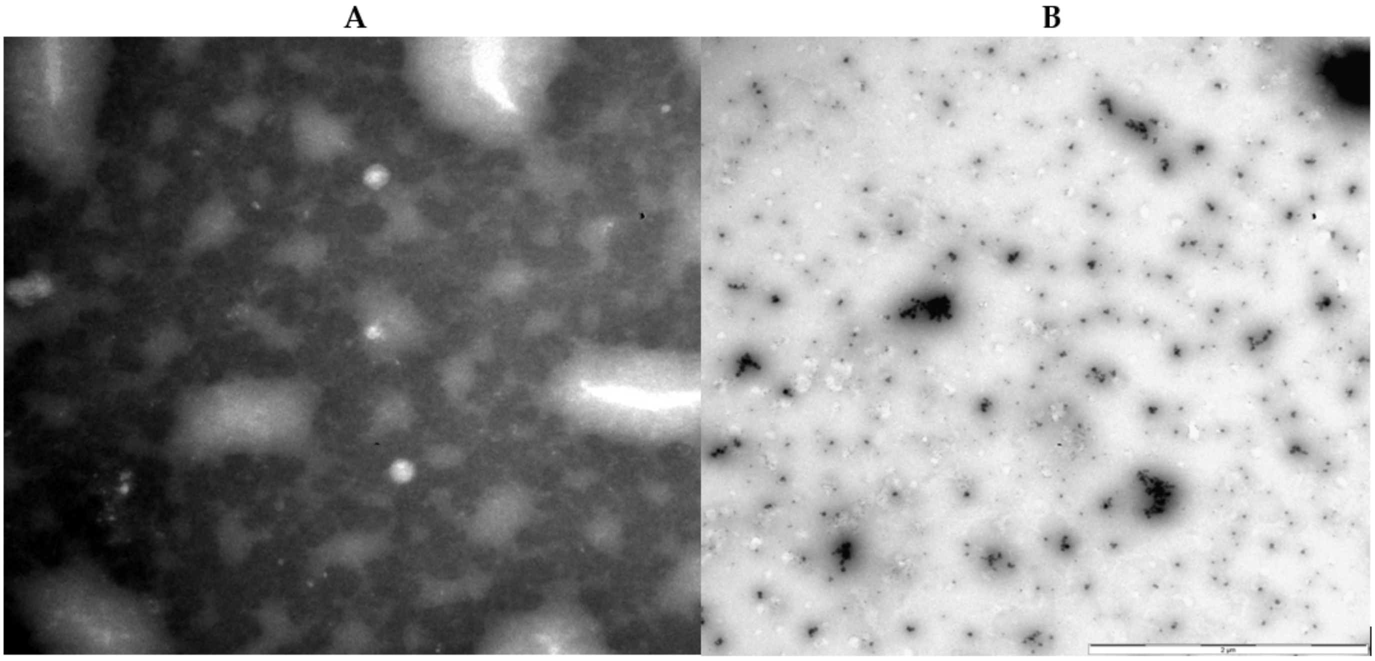

Supplementary Figure S5. Electron microscopy analyses of recombinant PCV2 Cap protein. A raw preparation of PCV2b strain DV6503 (positive control, Panel A) and recombinant, purified PCV2 Cap protein (Panel B) were submitted to negative staining on an electron microscope grid and analyzed by transmission electron microscopy (TEM). Panel A: presence of viral particles referable to Circovirus. Panel B: no particles referable to virions or VLPs were detected.

References

1. Walravens K, Wellemans V, Weynants V, Boelaert F, deBergeyck V, Letesson JJ, Huygen K, Godfroid J. Analysis of the antigen-specific IFN-gamma producing T-cell subsets in cattle experimentally infected with Mycobacterium bovis. *Vet Immunol Immunopathol* **2002**, 84(1-2):29-41.
2. Guarneri F., Tresoldi ET, Sarli G, Boniotti MB., Lelli D, Barbieri I, Bacci B, D'Annunzio G, Amadori M. Dataset of immune responses induced in swine by an inactivated Porcine Circovirus 2b vaccine. *Data Brief.* **2021**, *35*, 106906.
